# Accouchées avec statut sérologique VIH inconnu à Lubumbashi, RD Congo: proportion et déterminants

**Published:** 2012-06-08

**Authors:** Albert Mwembo-Tambwe A Nkoy, Prosper Kalenga Muenze Kayamba, Philippe Donnen, Faustin Chenge Mukalenge, Perrine Humblet, Michèle Dramaix, Pierre Buekens

**Affiliations:** 1 Département de Gynécologie et Obstétrique, Faculté de médecine de l'Université de Lubumbashi, RD Congo; 2 Ecole de Santé Publique de l'Université de l'Université de Lubumbashi, RD Congo; 3 Ecole de Santé Publique de l'Université libre de Bruxelles, Belgique; 4 School of Public Health and Tropical Medicine, Tulane University, New Orleans, Louisiana, USA

**Keywords:** VIH, Prévention de la transmission mère-enfant, statut, Congo

## Abstract

**Introduction:**

Beaucoup d'enfants vivant avec le VIH ont été infectés par leurs mères. Pour prévenir la transmission verticale les femmes doivent d'abord connaître leur statut sérologique VIH.L'objectif de cette étude était de déterminer la proportion de statut VIH inconnu à la naissance et d'identifier les facteurs associés.

**Méthodes:**

C'est une étude transversale réalisée dans 10 structures sanitaires de Lubumbashi de Juin à Septembre 2010. La taille de l’échantillon était de 602 accouchées. Les statistiques descriptives usuelles et la régression logistique ont été utilisées.

**Résultats:**

Parmi les accouchées, 52,5% ignoraient leur statut sérologique. Parmi elles, 62,9% accepteraient de faire le test VIH à la maternité. La proportion des femmes avec un statut sérologique VIH inconnu était significativement plus élevée chez celles qui n'avaient pas suivi de CPN (Odds Ratio ajusté (ORa) = 5,8; Intervalle de Confiance (IC) 95%: 1,7-19,8); chez celles qui avaient un bas niveau d'instruction (ORa = 1,5; IC 95%: 1,1-2,1) et chez celles qui ne savaient pas que la transmission verticale du VIH pouvaient se faire au moment de l'accouchement (ORa = 1,5; IC 95%: 1,0-2,4).

**Conclusion:**

La proportion de femmes qui accouchent sans connaître leur statut sérologique au VIH est encore importante, malgré le fait que le dépistage du VIH soit proposé lors des CPN. Dans les zones à haute séroprévalence de VIH, aucune femme ne devrait accoucher sans être dépistée au VIH. Ce serait une opportunité manquée.

## Introduction

La transmission de l'infection au VIH de la mère à l'enfant constitue un défi mondial [1, 2]. La majorité des enfants contaminés par le VIH se retrouve dans les pays en développement [2]. Pourtant des interventions efficaces ont pu réduire cette transmission à moins de 2% dans les pays développés [3–5]. Pour bénéficier de toutes ces interventions les femmes doivent d'abord connaître leur statut sérologique au VIH par le dépistage volontaire [2, 3].

En Afrique, les services de dépistage du VIH ont été introduits au niveau des Consultations Prénatales (CPN), depuis quand? Cependant, la couverture de ces services demeure insuffisante [6]. Par conséquent, beaucoup de femmes accouchent encore sans connaître leur statut sérologique au VIH et ne peuvent pas bénéficier des mesures de prévention adéquates dans ce cadre.

En République Démocratique du Congo (RDC), le problème posé par la transmission mère-enfant (TME) est préoccupant. Le nombre de nouveaux cas de VIH pédiatriques est de l'ordre de 28461 par an [7]. Pour lutter contre la transmission verticale, la politique nationale a intégré la Prévention de la Transmission du VIH de la Mère à l'Enfant (PTME) dans le paquet d'activités de la CPN. Comme dans d'autres pays, la couverture reste toujours insuffisante [8]. A Lubumbashi, dans le Sud-Est du pays, la prévalence du VIH chez les femmes enceintes est de 4,6% [7]. En 2007, l'acceptabilité globale (le produit de l'acceptation du pré-test, test et post- test) du dépistage du VIH aux CPN était de 33,7% chez les femmes enceintes à l'hôpital général de référence de Kenya du district sanitaire de Lubumbashi. Notons que toutes les femmes enceintes ne suivent pas correctement les séances de CPN [9]. Il existe donc un nombre élevé d'accouchées au statut sérologique VIH méconnu. Par conséquent, des accouchements à forte potentialité infectieuse au VIH ont encore lieu à Lubumbashi, alors que cela pourrait être évité. Aucune étude n'a été encore menée dans les maternités de Lubumbashi pour connaître le statut sérologique au VIH des accouchées. Ainsi, l'objectif de cette étude était de déterminer la proportion des accouchées au statut sérologique inconnu pour le VIH et d'identifier les facteurs qui y sont associés.

## Méthodes

C'est une étude observationnelle transversale descriptive réalisée dans les structures sanitaires de Lubumbashi du 26 Juin au 6 Septembre 2010.

Une enquête a été menée dans 10 dont 7 avec un service de Prévention pour la Transmission du VIH de la Mère à l'Enfant (PTME) intégré. Ces dernières ont été sélectionnées à par choix raisonné sur base du nombre d'accouchements élevé qui y sont réalisés et de leur localisation géographique différente. Par la suite au sein de chaque structure sélectionnée, un échantillon systématique a été prélevé.

Considérant qu'environ 50% d'accouchée ne connaissent pas leur statut sérologique dans notre milieu, avec une précision de 4%; la taille de l’échantillon retenue était de 602 parturientes.

Toute femme dont la grossesse avait au moins 28 semaines d'aménorrhée, ayant accouché depuis moins de 24 heures et ayant donné son consentement par écrit était éligible pour l’étude. Ainsi 1205 accouchées éligibles ont constitué la base de sondage à partir de la quelle le pas de sondage choisi était de 2.

Les données ont été recueillies par trois enquêtrices formées à l'aide d'un questionnaire structuré. Celui-ci comprenait les variables relatives aux informations sociodémographiques (l’âge maternel, le niveau d’étude, le statut matrimonial, l'emploi et le niveau socio-économique), la parité, la morbidité obstétricale antérieure (un accouchement prématuré, un avortement,un mort-né,un faible poids de naissance et une césarienne), suivi des consultations prénatales(au moins une fois) et les questions relatives au dépistage du VIH (avoir fait un dépistage du VIH au cours des dernières CPN), à la connaissance de la transmission du VIH de la mère à l'enfant pendant l'accouchement et l'allaitement. L'appartenance institutionnelle de la maternité et la présence d'un service de PTME ont été relevées.

Le niveau socio-économique a été apprécié à partir du calcul de l'indice de pauvreté tel que défini dans une enquête réalisée en RD Congo en 2001, le Multiple Indicator Cluster Survey (MICS2) [10]. C'est une mesure composée des caractéristiques des ménages: matériau du sol, nature du toit et des murs, biens appartenant au ménage, statut d'occupation du logement; les disponibilités et la durée des réserves alimentaires. Cette mesure de 36 points au total, nous a permis de regrouper les accouchées en niveaux socio-économiques faible (≤ 20 points), moyen (21-24 points) et élevé (≥ 25 points) en fonction de la distribution.

Le niveau d'instruction a été apprécié à partir du nombre d'années d’études accomplies par l'accouchée. Les accouchées on été regroupées en niveau d'instruction bas (≤ 9 année d’études), moyen (10-12 année d’études) et élevé (≥ 13 année d’études).

Cette étude a été autorisée par le comité d’éthique de l'université de Lubumbashi. Les autorités sanitaires ont donné leur accord par écrit.

Pour l'analyse des données, les statistiques descriptives usuelles ont été utilisées, ainsi qu'une mesure d'association (Odds Ratio) entre la connaissance du statut sérologique au VIH (à partir d'un dépistage du VIH fait lors des CPN) comme variable dépendante et les variables indépendantes suivantes: le suivi des CPN (facteur principal), les caractéristiques sociodémographiques, la parité, la morbidité obstétricale antérieure et la connaissance de la transmission verticale du VIH par l'accouchée (confondantes potentielles). Une régression logistique de la connaissance du statut sérologique au VIH a été réalisée dans une approche explicative. Pour vérifier l'adéquation du modèle final de régression, le test d'ajustement de Hosmer et Lemeshow a été appliqué. Le seuil de signification a été fixé à 0,05 et les intervalles de confiance à 95%. Les données ont été encodées dans Epi-info 3.4.1. 2007 et traitées à l'aide du logiciel STATA version 11.

## Résultats

Du Tableau 1, il se dégage que 2,8% des accouchées étaient des adolescentes (13-17 ans) et la grande majorité des femmes était mariée. Les accouchées de niveau d'instruction moyen représentaient 46,2%. Il convient de signaler que 48,4% d'accouchées ont été vues moins de 4 fois en Consultations Prénatales (CPN) (Tableau 1).

**Tableau 1:** Caractéristiques sociodémographiques des accouchées à Lubumbashi en 2010

Paramètres	n = 602	%
Age de l'accouchée		
Adolescentes (13-17 ans)	17	2,8
Adulte (≥18 ans)	585	97,2
**Etat matrimonial**		
Mariée	543	90,2
Non mariée	59	9,8
**Niveau d'instruction**		
Bas	279	46,3
Moyen	278	46,2
Elevé	45	7,5
**Niveau économique**		
Faible	283	47,0
Moyen	204	33,9
Elevé	115	19,1
**Emploi (un contrat de travail rémunérateur)**		
Oui	43	7,2
Non	555	92,8

Une proportion de 52,5% (IC 95%: 48,4-56,4%) des accouchées ne connaissaient pas leur statut sérologique au VIH.

Parmi celles qui connaissaient leur statut, les modalités du dépistage du VIH ont été les suivantes: 19 (6,6%) cas le test a été imposé à la gestante par le prestataire de CPN; dans 24 (8,4%) cas, il a été demandé par les accouchées elles-mêmes lors de leur CPN et dans 243 (85%) cas, il a été proposé par le prestataire et accepté par la gestante.

Selon les déclarations des accouchées, les causes majeures qui ont empêché la réalisation du dépistage du VIH étaient: le dépistage du VIH n'a pas été proposé par les prestataires lors des CPN (26,2%), le manque d'information (16,5%), la fidélité du couple (13,6%) et le manque d'intérêt pour ce test (12,3%) (Tableau 2).

**Tableau 2:** Causes ayant empêché la réalisation du dépistage du VIH chez les accouchées à Lubumbashi en 2010

Causes *	n = 316	%
Test non proposé par les prestataires des CPN†	83	26,2
Manque d'information	52	16,5
Couple fidèle et confiant	43	13,6
Test sans intérêt pour l'accouchée	39	12,3
Test déjà fait antérieurement ¥	22	7,0
Confiant en son statut sérologique (VIH négatif)	21	6,6
Sans raison	20	6,3
Manque d'argent	8	2,5
Service non disponible dans le site de CPN	8	2,5
Irrégularité aux CPN	5	1,6
Dépendance de la décision du mari	4	1,3
Peur de connaître sa sérologie VIH	4	1,3
Mauvaise organisation du service de PTME	3	1,0
CPN non suivies	2	0,6
Mauvaise communication entre le prestataire et la cliente	2	0,6

* L'interviewée devrait citer une seule cause (la plus importante pour elle).

† CPN: Consultation Prénatales.

¥ Test déjà fait antérieurement signifie: avant le mariage ou cette grossesse ou dans une autre circonstance.

§ PTME : Prévention de la Transmission du VIH de la Mère à l'Enfant

Du Tableau 3, il se dégage que la proportion des accouchées avec un statut sérologique inconnu au VIH était significativement plus élevée chez celles qui n'avaient pas suivi de CPN, chez celles de bas niveau d'instruction et chez celles qui ne savaient pas que la transmission verticale du VIH se faisait au moment de l'accouchement.

**Tableau 3:** Facteurs associés au statut sérologique VIH inconnu chez les accouchées à Lubumbashi en 2010

Facteurs	Statut sérologique VIH Inconnu (%)	OR brut (IC à 95%)	OR ajusté* (IC à 95%)
Age de l'accouchée			
Adulte (n = 585)	52,5	1,0	1,0
Adolescentes (13-17 ans) (n = 17)	52,9	1,0 (0,3-3,1)	1,1 (0,4-3,1)
**Etat matrimonial**			
Mariée (n = 543)	52,3	1,0	1,0
Non mariée (n = 59)	50,0	0,9 (0,5-1,7)	0,9 (0,4-1,6)
**Niveau d'instruction**			
Moyen et supérieur (n = 323)	46,8	1,0	1,0
Bas (n = 279)	59,1	1,6 (1,2-2,3)	1,5 (1,1-2,1)
**Niveau socioéconomique**			
Moyen et élevé (n = 319)	49,2	1,0	1,0
Faible (n = 283)	56,2	1,3 (0,9-1,8)	1,1 (0,7-1,5)
**Morbidité obstétricale antérieure †**			
Oui (n = 391)	51,9	1,0	1,0
Non (n = 211)	53,6	1,1 (0,7-1,5)	1,0 (0,7-1,4)
Parité (nombre d'enfant)			
≥ 3 (n = 364)	54,4	1,0	1,0
≤ 2 (=238)	49,6	0,8 (0,6-1,2)	0,8 (0,6-1,2)
**Suivi des CPN (au moins une fois)**			
Oui (n = 575)	50,8	1,0	1,0
Non (n = 27)	88,9	7,8 (2,3-40,1)	5,8 (1,7-19,8)
**Connaissance de la transmission verticale du VIH pendant l'accouchement**			
Oui (n = 446)	48,7	1,0	1,0
Non (n = 156)	63,5	1,8 (1,2-2,7)	1,5 (1,0-2,4)
**Connaissance de la transmission verticale du VIH pendant l'allaitement**			
Oui (n = 424)	49,8	1,0	1,0
Non (n = 178)	59,0	1,5 (1,0-2,1)	1,2 (0,7-1,7)

* OR ajutés par rapport l’âge, état matrimonial, niveau d'instruction, niveau socioéconomique, morbidité obstétricale antérieure et parité et le niveau de connaissance de la PTME de l'accouchée au moyen de la régression logistique; IC: Intervalle de Confiance.

† Morbidité obstétricale antérieure: un accouchement prématuré, un avortement, un mort-né, un faible poids de naissance et une césarienne.

Sur 316 accouchées qui n'avaient pas fait le dépistage du VIH lors des CPN, 62,7% (IC 95%: 57,3-68,3%) étaient disposées à le faire à la maternité si on le leur proposait.

## Discussion

Parmi les accouchées interrogées, 52,5% ne connaissent pas leur statut sérologique au VIH. Cette proportion est supérieure à celle observée dans 6 hôpitaux et centres de santé du Zimbabwe en 2006 (45,2%)[11]. Et pourtant, pour réduire les risques de transmission verticale du VIH, les mères doivent connaître leur statut vis à vis du VIH. Le postulat sous-jacent est que les femmes enceintes qui se savent infectées par le VIH ont plus de chance d’être motivées à se soucier de leur santé et celle de leurs futurs enfants. La méconnaissance du statut sérologique de la femme enceinte jusqu’à l'accouchement, constitue donc un obstacle à la lutte contre la transmission verticale du VIH. Car le test de dépistage du VIH est le point d'entrée pour les interventions spécifiques de la PTME. A titre d'exemple, l'OMS [12] recommande que: «toutes les femmes enceintes infectées par le VIH qui n'ont pas besoin de traitement pour leur propre santé ont besoin d'une stratégie efficace de prophylaxie par les antirétroviraux pour prévenir la transmission du VIH à leurs enfants. Cette prophylaxie doit commencer dès la 14ème semaine de grossesse ou dès que possible chez les femmes qui se présentent tard au cours de la grossesse, au cours du travail ou de l'accouchement ». A l'heure actuelle, aucune femme ne devrait accoucher dans les zones à haute prévalence sans être dépistée au VIH. Ce serait une « opportunité manquée ».

Plusieurs facteurs entrent en ligne de compte pour expliquer cette proportion élevée de femmes au statut sérologique VIH inconnu. Certains sont liés au service et d'autres à la communauté ou à la femme enceinte elle-même. Ces facteurs sont associés à l'acceptabilité du dépistage du VIH aux CPN. L'acceptation du test est faible en cas de longue période d'attente avant les consultations et lorsque le nombre de conseillers est insuffisant [13, 14]. Les modalités du dépistage jouent également un rôle important. Dans 8% des cas, il a été demandé par les accouchées elles-mêmes lors de CPN. Lorsque le dépistage est rarement demandé, peu de personnes connaîtront leur statut sérologique au VIH [15]. Les études récentes ont montré une corrélation entre le dépistage du VIH chez les gestantes et la stratégie utilisée. Les programmes qui attendent que la demande vienne de la femme enceinte (Opt-in) présentent des faibles proportions de dépistage par rapport à ceux qui proposent systématiquement le test à toutes les femmes enceintes aux CPN de routine (Opt-out) [16, 17]. Cependant, la proposition devrait se faire de manière à permettre à la femme enceinte d'accepter librement ce test. Dans le même ordre d'idées, un service de mauvaise qualité peut conduire les femmes enceintes à abandonner les consultations après les résultats du dépistage et à ne plus être couvertes par les étapes suivantes [14]. C'est le cas d'une communication des résultats du test longtemps après le prélèvement de l’échantillon par exemple. Dans le cas de la ville de Lubumbashi, le counseling est dévolu à des personnes formées, qui du reste sont relativement peu nombreuses. Ceci est dû au fait que ces services ont été introduits verticalement dans les structures par des ONG, en limitant le nombre de personnes à former. Vu la fréquentation élevée des services, il existe des risques de surcharge et de fatigue pouvant altérer la qualité du travail. Pour palier à cette situation, le gouvernement devrait y investir suffisamment pour former les prestataires de soins en salle de travail.

Selon les accouchées interrogées (Tableau 2), parmi les causes ayant empêché la réalisation du dépistage du VIH nous notons: le teste non proposé par les prestataires des CPN, le manque d'information, la fidélité du couple et le manque d'intérêt de ce test. Certaines de ces causes sont similaires à celles avancées par Perez et al [11], Moth et al [13], Kalichman et al [18] et Muchedzi et al [19] dans leurs études respectives. Ceci montre d'une part la nécessité d'une sensibilisation de la population centrée sur la PTME et d'autre part, une organisation de service pouvant augmenter son accessibilité géographique, socioculturelle et financière. En ce qui concerne les facteurs favorisant une bonne acceptabilité du dépistage du VIH, plusieurs auteurs retiennent la perception du bénéfice de l'examen, la confidentialité des résultats, la disponibilité des antirétroviraux, une bonne information sur le Sida, l'existence des services de PTME dans le milieu et l'approche du dépistage adaptée au contexte [18, 20]. De manière significative, dans cette étude, le statut sérologique du VIH inconnu était élevé chez celles qui n'avaient pas suivi de CPN, les moins instruites et celles qui ne savaient pas que la transmission verticale du VIH se faisait au moment de l'accouchement (Tableau 3). Des résultats semblables ont été obtenus par Perez et al [16]. Un niveau d’étude secondaire et l'existence d'un service de PTME à l'endroit des CPN étaient associés positivement à l'acceptabilité du test de dépistage au VIH. Pour expliquer ces observations, le niveau socioéconomique et d'instruction bas sont des obstacles à l'utilisation de service de santé maternelle et infantile [21]. Notons que dans notre série, 51,6% des accouchées ont bénéficié de 4 CPN au moins. Il est important pour le prestataire d'avoir des bonnes connaissances sur les soins prénataux focalisés permettant d'assurer le bon suivi du couple mère-enfant. Dans cette approche, l'accent est mis sur la qualité des consultations plutôt que sur le nombre. Le prestataire doit aider les femmes enceintes et leurs partenaires sexuels masculins à prévenir la transmission verticale du VIH.

Au demeurant, parmi les accouchées dont le statut sérologique du VIH était inconnu, 62,9% ont déclaré qu'elles accepteraient le dépistage du VIH même à la maternité. Ceci prouve la disposition des femmes à connaître leur statut sérologique. Il appartient donc, au service de répondre à ce besoin. En salle de travail, un dépistage de rattrapage du VIH pouvait être proposé à cette catégorie de femmes [22].

### Limite de l’étude

Notre enquête a porté seulement sur les accouchées présentes au moment de l'investigation dans les maternités. Cela constitue une faiblesse liée à la nature d'une étude transversale. Malgré cette limite, c'est le premier travail réalisé dans notre milieu, qui détermine l'importante proportion d'accouchées qui ne connaissent pas leur statut sérologique VIH et les déterminants de ce statut, à cette époque o[ugrave] la transmission mère-enfant doit être éradiquée.

## Conclusion

La proportion des femmes qui accouchent sans connaître leur statut sérologique au VIH est encore importante, malgré le fait que le dépistage du VIH soit proposé lors des CPN. A l'heure actuelle, aucune femme ne devrait accoucher dans les zones à haute prévalence sans être dépistée au VIH. Ce serait une « opportunité manquée ».

## Remerciements

Les auteurs remercient la Coopération Technique Belge (CTB) pour avoir financé cette étude et les autorités sanitaires de la ville de Lubumbashi pour leur soutien.
